# Lipopolysaccharide Induces Inflammatory Hyperalgesia Triggering a TLR4/MyD88-Dependent Cytokine Cascade in the Mice Paw

**DOI:** 10.1371/journal.pone.0090013

**Published:** 2014-03-03

**Authors:** Igor L. Calil, Ana C. Zarpelon, Ana T. G. Guerrero, Jose C. Alves-Filho, Sergio H. Ferreira, Fernando Q. Cunha, Thiago M. Cunha, Waldiceu A. Verri

**Affiliations:** 1 Department of Pharmacology, Ribeirão Preto Medical School, University of São Paulo, Ribeirão Preto, São Paulo, Brazil; 2 Departamento de Patologia, Centro de Ciências Biologicas, Universidade Estadual de Londrina, Londrina, Paraná, Brazil; 3 FIOCRUZ Mato Grosso do Sul, Campo Grande, Mato Grosso do Sul, Brazil; SRI International, United States of America

## Abstract

Inflammatory pain can be triggered by different stimuli, such as trauma, radiation, antigen and infection. In a model of inflammatory pain caused by infection, injection in the mice paw of lipopolysaccharide (LPS), a Toll-like receptor 4 (TLR4) agonist, produces mechanical hyperalgesia. We identify here the TLR4 linked signaling pathways that elicit this response. Firstly, LPS paw injection in wild type (WT) mice produced mechanical hyperalgesia that was not altered in TRIF^-/-^ mice. On the other hand, this response was absent in TLR4 mutant and MyD88 null mice and reduced in TNFR1 null mice. Either an IL-1 receptor antagonist, anti-KC/CXCL1 antibody, indomethacin or guanethidine injection also lessened this response. Moreover, LPS-induced time dependent increases in TNF-α, KC/CXCL1 and IL-1β expression in the mice paw, which were absent in TLR4 mutant and MyD88 null mice. Furthermore, in TNFR1 deficient mice, the LPS-induced rises in KC/CXCL1 and IL-1β release were less than in their wild type counterpart. LPS also induced increase of myeloperoxidase activity in the paw skin, which was inhibited in TLR4 mutant and MyD88 null mice, and not altered in TRIF^-/-^ mice. These results suggest that LPS-induced inflammatory pain in mice is solely dependent on the TLR4/MyD88 rather than the TLR4/TRIF signaling pathway. This pathway triggers pronociceptive cytokine TNF-α release that in turn mediates rises in KC/CXCL1 and IL-1β expression. Finally, these cytokines might be involved in stimulating production of directly-acting hyperalgesic mediators such as prostaglandins and sympathomimetic amine.

## Introduction

Inflammatory pain is a common symptom of all types of inflammatory diseases. Among the mechanisms involved in the genesis of inflammatory pain, the sensitization of pain receptor (peripheral sensitization) is a central event. This event leads to an increase in pain sensation, which is clinically referred to as hyperalgesia. C-Polymodal, high-threshold nociceptors or thin-myelinated fibers have long been associated with inflammatory hyperalgesia [1], [2]. Nociceptor sensitization is caused by the action of inflammatory mediators such as prostaglandins and sympathomimetic amines, which act directly on their cognate receptors expressed by primary nociceptive fibers [3], [4].

It is well known that the release of these direct-acting hyperalgesic mediators is generally preceded by a cascade of cytokines in rats and mice [3], [5]. For instance, in mice, carrageenin induces a concomitant release of tumor necrosis factor (TNF-α) and keratinocyte-derived chemokine (KC/CXCL1). Both mediators stimulate the subsequent release of interleukin-1β (IL-1β), which in turn induces the production of prostaglandins. Furthermore, KC/CXCL1 also stimulates the sympathetic component of inflammatory hyperalgesia [5]. It was also demonstrated that during antigen-induced inflammation in immunized mice the same cytokines participate in the genesis of mechanical hyperalgesia [6].

Toll-like receptor 4 (TLR4) is a transmembrane receptor protein with extracellular leucine-rich repeated domains and a cytoplasmic signaling domain especially involved in the activation of innate immune response. It recognizes both pathogen-associated molecular patterns (PAMPs) and endogenous danger signals (DAMPs) and signals via the Toll/Interleukin-1 receptor (TIR) domain through MyD88- and TRIF (Toll-IL-1R-containing adaptor inducing IFN-β)-dependent pathways. Normally, TLR4 activation either by PAMPs or DAMPs results in a profound proinflammatory response in a NF-κB-dependent manner [7]. Lipopolysaccharide (LPS) is a well-recognized TLR4 agonist that is a component of gram-negative bacterial walls. One of the uses of this agonist is to analyze TLR4 functional involvement in inducing acute and chronic pain [6], [8], [9]. This model is also a widely used model of inflammation [6], [7], [8], [9], [10]. For instance, we have shown that intraplantar (i.pl.) injection of LPS into the rat hindpaw induces a dose-dependent mechanical inflammatory hyperalgesia [10]. However, it is still unclear which cell signaling pathways elicit this response. We report here on studies identifying cell-signaling pathways mediating LPS-induced hyperalgesia.

## Materials and Methods

### Ethics statement

All experiments were conducted in accordance with the National Institute of Health prescribed guidelines on experimental animal welfare and met with the approval of the Ethics Committee of the Ribeirão Preto Medical School, University of São Paulo.

### Mice

Male C57BL/6 and SV129 wild type (WT) mice, MyD88 null mice ^(-/-)^, TNFR1^-/-^ (20-25 g) were used to investigate cell signaling pathways mediating TLR4-induced hyperalgesia. The TLR4 mutant (C3H/HeJ) mice and their littermates C3H/HePas were used, which carry a dominant-negative mutation in the TLR4 cytoplasmic domain. TNFR1^-/-^, C3H/HePas and C3H/HeJ mice were obtained from Jackson Laboratories (Bar Harbor, ME, USA). MyD88^-/-^, TLR2^-/-^ and TRIF^-/-^ were generated by S. Akira (Osaka University, Japan) and they were provided by R.T. Gazzinelli. TNFR1^-/-^, MyD88^-/-^, TLR2^-/-^ mice were backcrossed for at least eight generations into the C57BL/6 background. TRIF^-/-^ mice were in a SV129 background. Mice were housed in the animal care facility at the Ribeirão Preto Medical School, University of Sao Paulo. Mice were taken to the testing room at least 1 h before experiments and were used once. All experiments were double blinded.

### Reagents

The following materials were obtained from the indicated sources. Murine recombinant TNFα, IL-1β and human recombinant IL-1ra were provided by the National Institute for Biological Standards and Control (South Mimms, Hertfordshire, U.K.). Recombinant murine KC/CXCL1 (catalog# 250-11) and anti-KC/CXCL1 antibody (catalog# 500-P115) were purchased from PeproTech (Rocky Hill, NJ, USA). Dopamine (catalog# H8502), guanethidine (catalog# G8520), LPS (catalog# L3024) and PGE_2_ (catalog# P5640) were purchased from Sigma- Aldrich, St. Louis, MO, USA) and indomethacin was donated by Prodome Química e Farmacêutica (Campinas, Brazil).

### Mechanical nociceptive paw test

Mechanical hyperalgesia was tested in mice as previously reported [11]. Mice were housed in acrylic cages (8×8×12 cm) with wire grid floors 15–30 min before the start of testing. The threshold of nociceptive responsiveness to mechanical stimuli applied to the right hindpaw was assessed using a dynamic plantar anesthesiometer (Model 37400, Ugo Basile, Milan, Italy), an electronic and automatic version of the von Frey test [12]. The test consisted of evoking a hindpaw flexion reflex with an automatic force transducer adapted with a 0.5 mm^2^ polypropylene tip. The investigator was trained to apply the tip to the lateral area of the incised hind paw with a gradual increase in pressure of 2 g/s. The gradual increase in pressure was performed in blinded experiments. The upper limit of pressure was 20 g. The end-point was characterized by the removal of the paw followed by clear flinching movements. After paw withdrawal, the intensity of the pressure was automatically recorded, and the final value for the response was obtained by averaging three measurements. The animals were tested before and after treatments. The results are expressed as the withdrawal threshold, in g. The withdrawal threshold was 7.1±0.2 g (mean ± S.E.M.; n = 20) before injection of the hyperalgesic agents.

### Cytokine measurements

At indicated times after the injection of inflammatory stimuli, animals were terminally anesthetized, and the skin tissues of the plantar region were collected from the LPS and saline (control) injected paws. The samples were triturated and homogenized in 500 µl of the appropriate buffer containing protease inhibitors followed by centrifugation for 10 min at 2,000 *g*. The supernatants were used to determine the levels of TNF-α (catalog# DY410), IL-1β (catalog# DY401) and KC/CXCL1 (catalog# DY453) by enzyme-linked immunosorbent assay (ELISA) using Duoset kits from R&D Systems [4]. Briefly, microtiter plates were coated overnight at 4°C with either an immunoaffinity-purified polyclonal sheep antibody against KC/CXCL1 (2 µg/mL), TNF-α (0.8 µg/mL) or IL-1β (4 µg/mL). After blocking the plates, recombinant murine KC/CXCL1, TNF-α or IL-1β standards at various dilutions and the samples were added in duplicate and then incubated overnight at 4°C. Either rabbit biotinylated immunoaffinity-purified antibody anti-KC/CXCL1-2 (200 ng/mL), anti-TNF-α (50 ng/ml) or anti-IL-1β (1.5 µg/ml) was added, followed by incubation at room temperature for 1 h. Finally, 100 µl of avidin-HRP (concentration depending on each kit; DAKO A/S, Denmark) were added to each well, and after 30 min the plates were washed and the color reagent OPD (200 µg/well; Sigma) was added. After 15 min, the reaction was stopped with 1M H_2_SO_4_ and the optical density (O.D.) read at 490 nm. The results are expressed as pg of each cytokine per paw. The control groups are indicated in the figure legends.

### Myeloperoxidase Activity Assay

LPS-induced leukocyte migration to the paw skin was evaluated using a myeloperoxidase kinetic-colorimetric assay. Tissue samples were collected in 50 mM K_2_HPO_4_ buffer (pH 6.0) containing 13.72 mM HTAB and stored at −20°C until assayed. The samples were homogenized using a Tissue-Tearor (Biospec), and then, homogenates were centrifuged (16,100 g, 2 min, 4°C) and the resulting supernatants assayed spectrophotometrically for myeloperoxidase activity determination at 450 nm (Victor^3^ 1420 multilabel counter). The myeloperoxidase activity of samples was compared to a standard curve of neutrophils and presented as the number of neutrophils per mg of tissue [13].

### Experimental protocols

In the first series of experiments, HePas mice received intraplantar (i.pl. – e.g. subcutaneous injection in the plantar surface of the paw) injection of LPS (30–300 ng/paw) or saline (20 µl), and HePas and Hej mice received i.pl. injection of LPS (100 ng/paw). Mechanical hyperalgesia was evaluated before and 1–24 h after LPS injection. In the second series of experiments, MyD88^+/+^ (C57BL/6 background), MyD88^-/-^ (C57BL/6 background), TRIF^+/+^ (129Sv background) and TRIF^-/-^ (129Sv background) mice received i.pl. injection of LPS (100 ng/paw) or saline. Mechanical hyperalgesia was evaluated 3 h (peak of mechanical hyperalgesia) after LPS injection. In the third series of experiments, LPS (100 ng/paw) was injected in TNFR1^+/+^ (C57BL/6 background) or TNFR1^-/-^ (C57BL/6 background) mice, and in C57BL/6 wild type mice were treated with anti-CXCL1 antibody (500 ng/paw, 5 min before LPS injection), IL-1ra (500 ng/paw, 5 min before LPS injection), indomethacin, (5 mg/kg, i.p. 30 min before LPS injection), guanethidine (30 mg/kg, s.c. 60 min before LPS injection) or indomethacin plus guanethidine (same doses). Mechanical hyperalgesia was evaluated 3 h after LPS injection. In the forth series of experiments, HePas and Hej mice received i.pl. injection of saline (20 µl), TNF-α (100 pg/paw), CXCL1 (20 ng/paw), IL-1β (1 ng/paw), PGE_2_ (100 ng/paw) or dopamine (10 µg/paw). Mechanical hyperalgesia was evaluated 3 h after i.pl. injection of hyperalgesic mediators. In the fifth series of experiments, LPS (100 ng/paw) or saline was injected into the HePas mice hindpaw and subcutaneous tissue samples were collected at 0.5, 1, 3 and 5 h for TNF-α, CXCL1/KC or IL-1β determination by ELISA. In the fifth and sixth series of experiments, LPS (100 ng/paw) or saline was also injected into the HePas, Hej, MyD88^+/+^ (C57BL/6 background) and MyD88^-/-^ (C57BL/6 background) mice hindpaw and after 3 h subcutaneous tissue samples were collected for TNF-α, CXCL1 or IL-1β determination by ELISA. In the seventh series of experiments, saline or LPS (100 ng/paw) was injected into the TNFR1^+/+^ (C57BL/6 background) or TNFR1^-/-^ (C57BL/6 background) mice hindpaw. At 3 h after LPS injection, the subcutaneous tissue samples were collected for CXCL1/KC, or IL-1β determination by ELISA. In the eighth series of experiments, LPS (100 ng/paw) or saline was injected in HePas, Hej, MyD88^+/+^ (C57BL/6 background), MyD88^-/-^ (C57BL/6 background), TRIF^+/+^ (129Sv background) and TRIF^-/-^ (129Sv background) and myeloperoxidase activity was evaluated after 5 h in paw skin samples.

### Statistical analyses

Results are presented as means ± S.E.M. and are representative of two separate experiments of 5 animals per group. Two-way analysis of variance (ANOVA) was used to compare the groups and doses at all times (curves) when the hyperalgesic responses were measured at different times after the stimulus injection. The analyzed factors were treatment regimens, time and time *versus* treatment interaction. When there was a significant time *versus* treatment interaction, one-way ANOVA followed by Bonferroni's *t* test was performed for each time. Alternatively, when the hyperalgesic responses were measured once after the stimulus injection, the differences between responses were evaluated by one-way ANOVA followed by Bonferroni's *t-*test. Statistical differences were considered to be significant at *P*<0.05.

## Results

### LPS induces a TLR4-dependent mechanical inflammatory hyperalgesia

**Figure 1:**
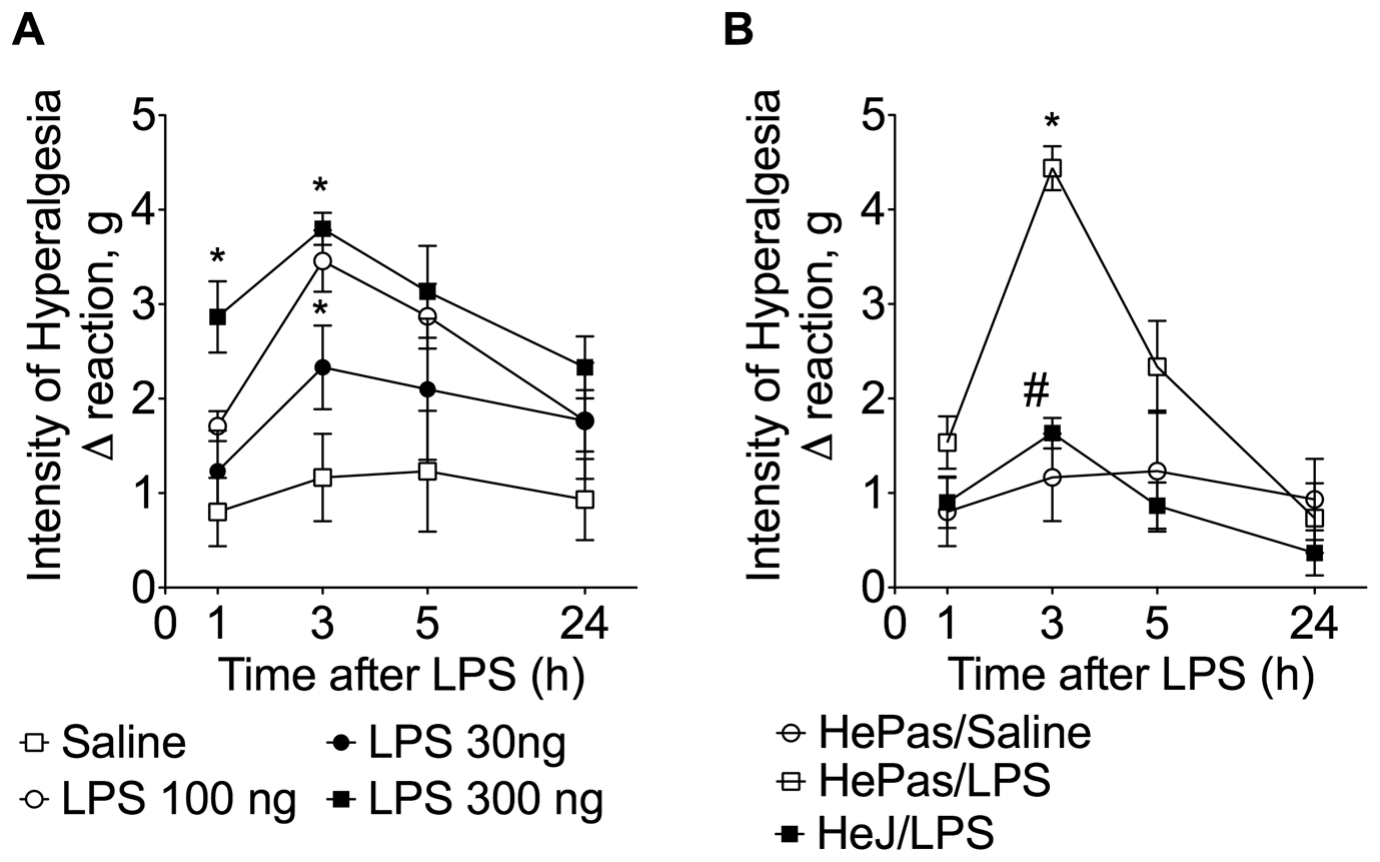
Role of TLR4 in LPS-induced mechanical inflammatory hyperalgesia in the mice paw. (**A**) HePas mice received intraplantar (i.pl.) injection of LPS (30–300 ng/paw) or saline (20 µl). (**B**) HePas and Hej mice received i.pl. injection of LPS (100 ng/paw). Mechanical hyperalgesia was evaluated 1–24 h after LPS injection. *P<0.05 compared with saline control; #*P*<0.05 compared with HePas injected with LPS (Panel C). One-way ANOVA followed by Tukey's t test.

Firstly, LPS dosage and its time dependent effects on hyperalgesia were evaluated in HePas mice (WT control). These mice received intraplantar (i.pl.) LPS injection (30–300 ng) or saline (25 µl). Mechanical hyperalgesia was evaluated until 24 h after injection (Fig. 1A). LPS induced mechanical hyperalgesia in a dose- and time-dependent manner (Fig. 1A). These responses were maximal at 3 h after LPS injection. No statistical differences in mechanical hyperalgesia were detected between LPS doses of 100 and 300 ng/paw (Figure 1A). Therefore, a 100 ng LPS dose was used for the following experiments. We determined whether or not LPS induces hyperalgesia in HeJ (TLR4 signalling deficient) mice (Fig. 1B). Even though this response was always induced at all times in HePas mice, it was not observed in HeJ mice. This difference indicates that TLR4-induced cell signalling activation is required for eliciting mice paw mechanical hyperalgesia.

### LPS induces mechanical hyperalgesia through MyD88 signalling pathway activation

**Figure 2:**
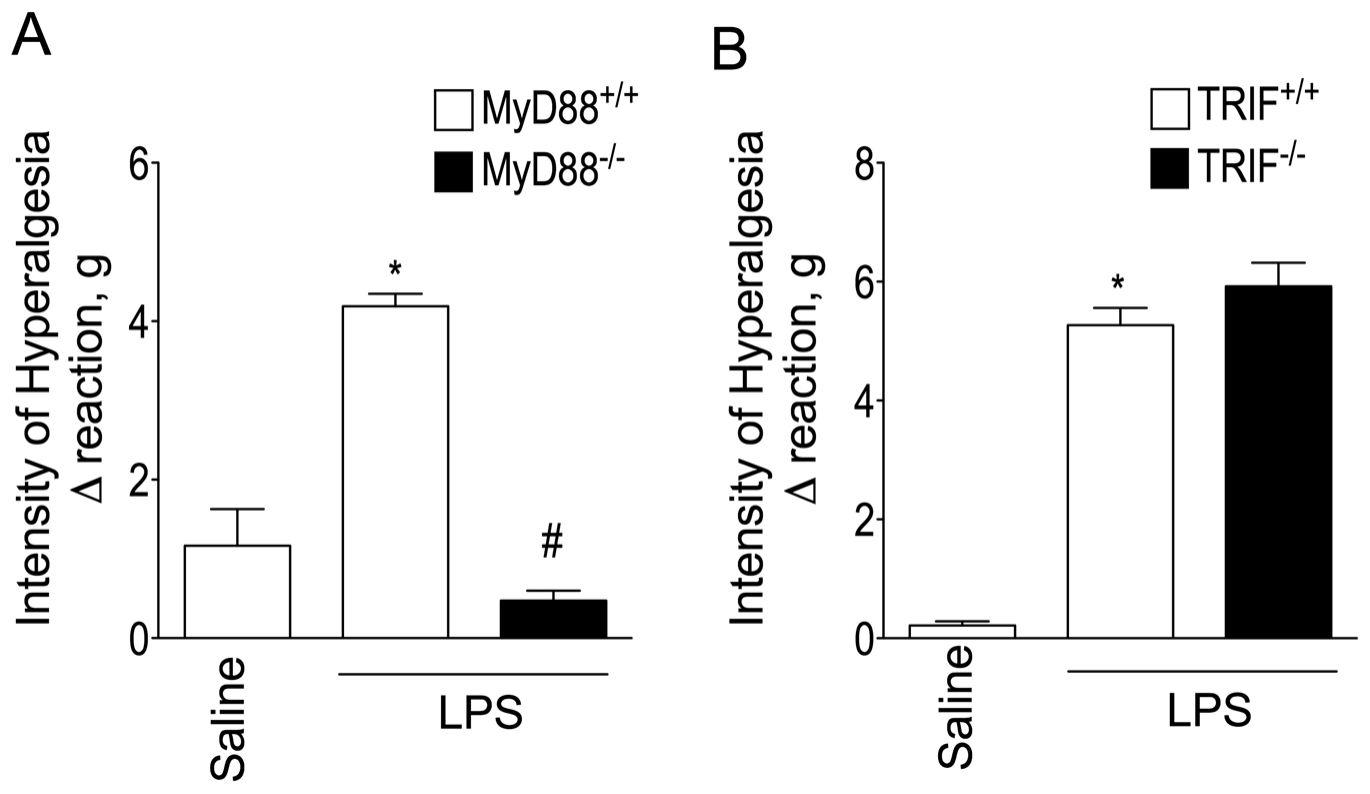
Role of MyD88 and TRIF signalling pathways in LPS-induced mechanical inflammatory hyperalgesia in the mice paw. (A) MyD88^+/+^ (C57BL/6 background) and MyD88^-/-^ (C57BL/6 background) mice received i.pl. injection of LPS (100 ng/paw) or saline. (B) TRIF^+/+^ (129Sv background) and TRIF^-/-^ (129Sv background) mice received i.pl. injection of LPS (100 ng/paw) or saline. Mechanical hyperalgesia was evaluated 3 h after LPS injection. *P<0.05 compared with saline control, #*P*<0.05 compared with MyD88^+/+^ or TRIF^+/+^ mice injected with LPS. One-way ANOVA followed by Tukey's t test.

TLR4 activation induces responses through MyD88- and TRIF-dependent signaling pathways [14], [15]. We delineated which of these pathways elicits increases in mechanical hyperalgesia induced by plantar injection of LPS. The mechanical hyperalgesic response occurring in WT (C57BL/6) mice was completely absent in MyD88^-/-^ mice (Figure 2A) while it was unaltered in TRIF^-/-^ mice compared to that obtained in the control group (SV129) (Figure 2B). These results suggest that MyD88 rather than TRIF-dependent signaling is solely responsible for eliciting LPS-induced mechanical hyperalgesia.

### Cytokines, prostaglandins and sympathetic amines mediate LPS-induced mechanical hyperalgesia in a TLR4/MyD88 signalling-dependent manner

**Figure 3:**
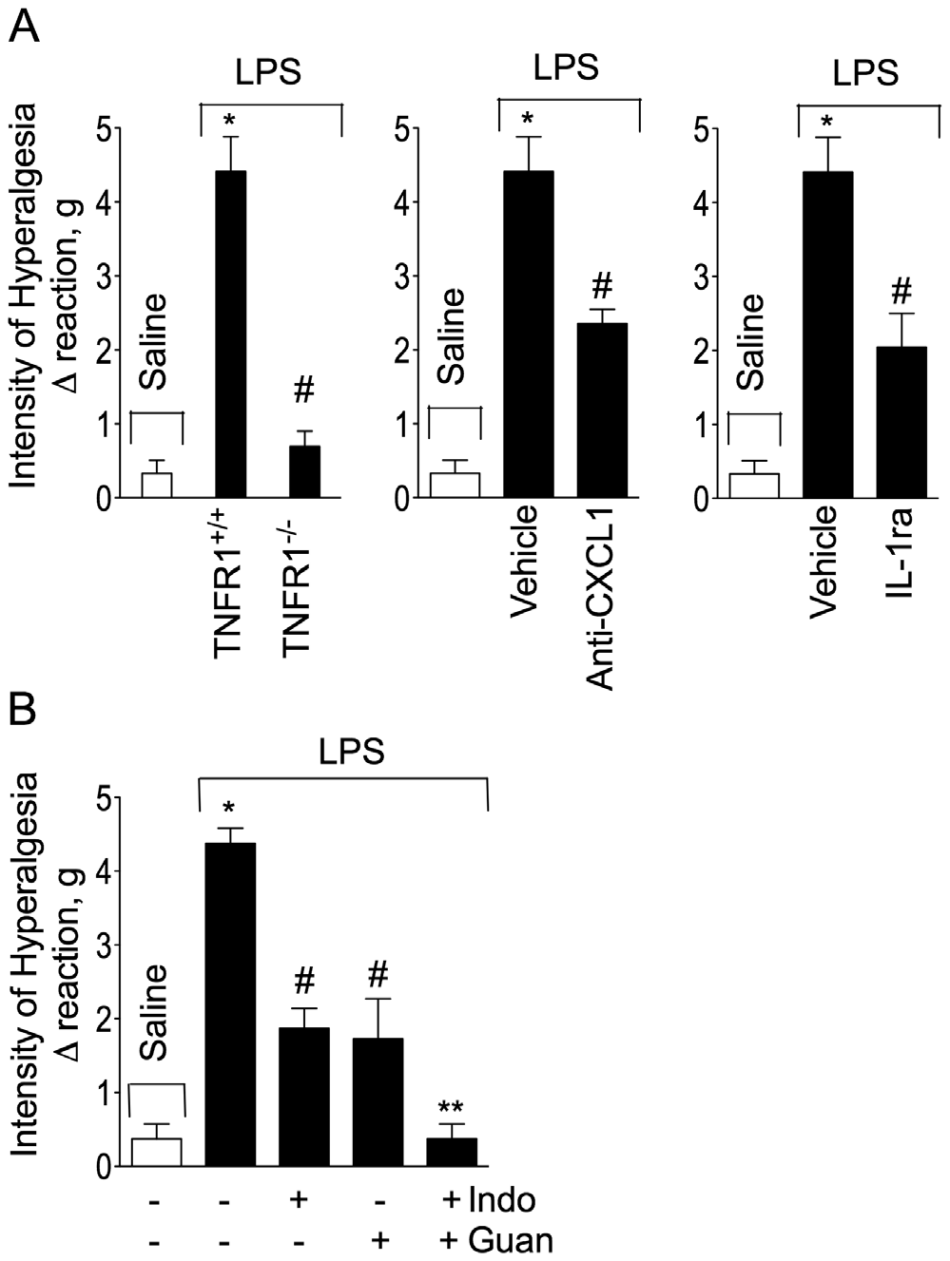
LPS-induced mechanical inflammatory hyperalgesia depends on cytokines, prostaglandins and sympathomimetic amines. (**A**) LPS (100 ng/paw) was injected in TNFR1^+/+^ (C57BL/6 background) or TNFR1^-/-^ (C57BL/6 background) mice. (**B**) C57BL/6 wild type mice were treated with anti-CXCL1 antibody (500 ng/paw, 5 min before LPS injection), or (**C**) IL-1ra (500 ng/paw, 5 min before LPS injection), or (**D**) indomethacin, (5 mg/kg, i.p. 30 min before LPS injection), guanethidine (30 mg/kg, s.c. 60 min before LPS injection) or indomethacin plus guanethidine (same doses). Mechanical hyperalgesia was evaluated 3 h after LPS injection. *P<0.05 compared with saline control, #*P*<0.05 compared with LPS control group; **P<0.05 compared with indomethacin or guanethidine treated group. One-way ANOVA followed by Tukey's t test.

The cytokines, prostaglandins and sympathomimetic amines involved in mediating the inflammatory component of mechanical hyperalgesia induced by i.pl. injection of LPS were evaluated. The different groups of mice included: a) TNFR1^-/-^ mice or mice pre-treated with IL-1Ra (500 ng i.pl. 5 min before LPS injection); b) anti-KC/CXCL1 antibody (500 ng i.pl. 5 min before LPS injection); c) indomethacin (5 mg/kg, i.p. 40 min before LPS injection); d) guanethidine (30 mg/Kg s.c 1 h before LPS injection), association of indomethacin plus guanethidine or vehicles. LPS-induced paw hyperalgesia was reduced by 80% in TNFR1 null mice compared with WT mice (Figure 3A). Local (paw) treatment of mice with IL-1Ra or anti-KC/CXCL1 reduced mechanical hyperalgesia by 44% and 29%, respectively (Figure 3A). Moreover, this response was reduced by indomethacin (48%), guanethidine (64%) whereas it was eliminated by coadministering both of these drugs (Figure 3B). These results suggest that TNF-α, IL-1β, KC/CXCL1, prostaglandins and sympathomimetic amines contribute to mediating LPS-induced mechanical inflammatory hyperalgesia in mice.

**Figure 4:**
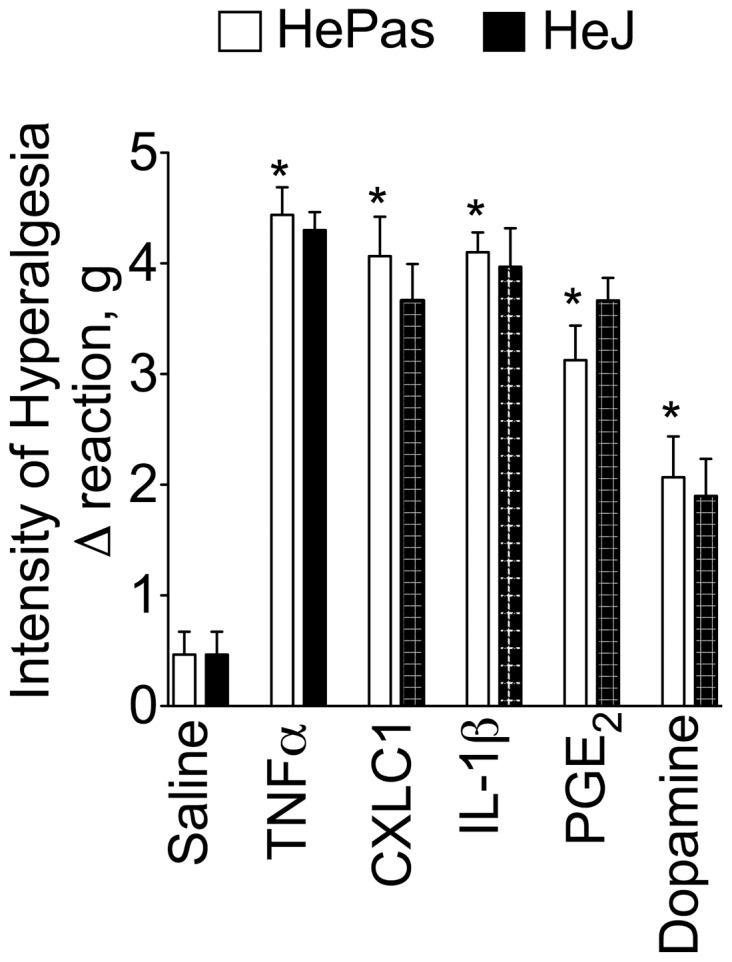
Cytokines- and direct acting mediators-induced mechanical hyperalgesia in HePas and Hej mice. HePas and Hej mice received i.pl. injection of saline (20 µl), TNF-α (100 pg/paw), CXCL1 (20 ng/paw), IL-1β (1 ng/paw), PGE_2_ (100 ng/paw) or dopamine (10 µg/paw). Mechanical hyperalgesia was evaluated 3 h after i.pl. injection of hyperalgesic mediators. *P<0.05 compared with saline control. One-way ANOVA followed by Tukey's t test.

To confirm TLR4 activation is upstream of the inflammatory mediator release, HePas and Hej mice received i.pl. injection of saline (25 µl), TNF-α (100 pg/paw), KC/CXCL1 (20 ng/paw), IL-1β (1 ng/paw), PGE_2_ (100 ng/paw) or dopamine (10 µg/paw) and mechanical hyperalgesia was evaluated 3 h after injection. All of these agents elicited hyperalgesia with similar magnitude in HePas and Hej mice (Fig. 4).

**Figure 5:**
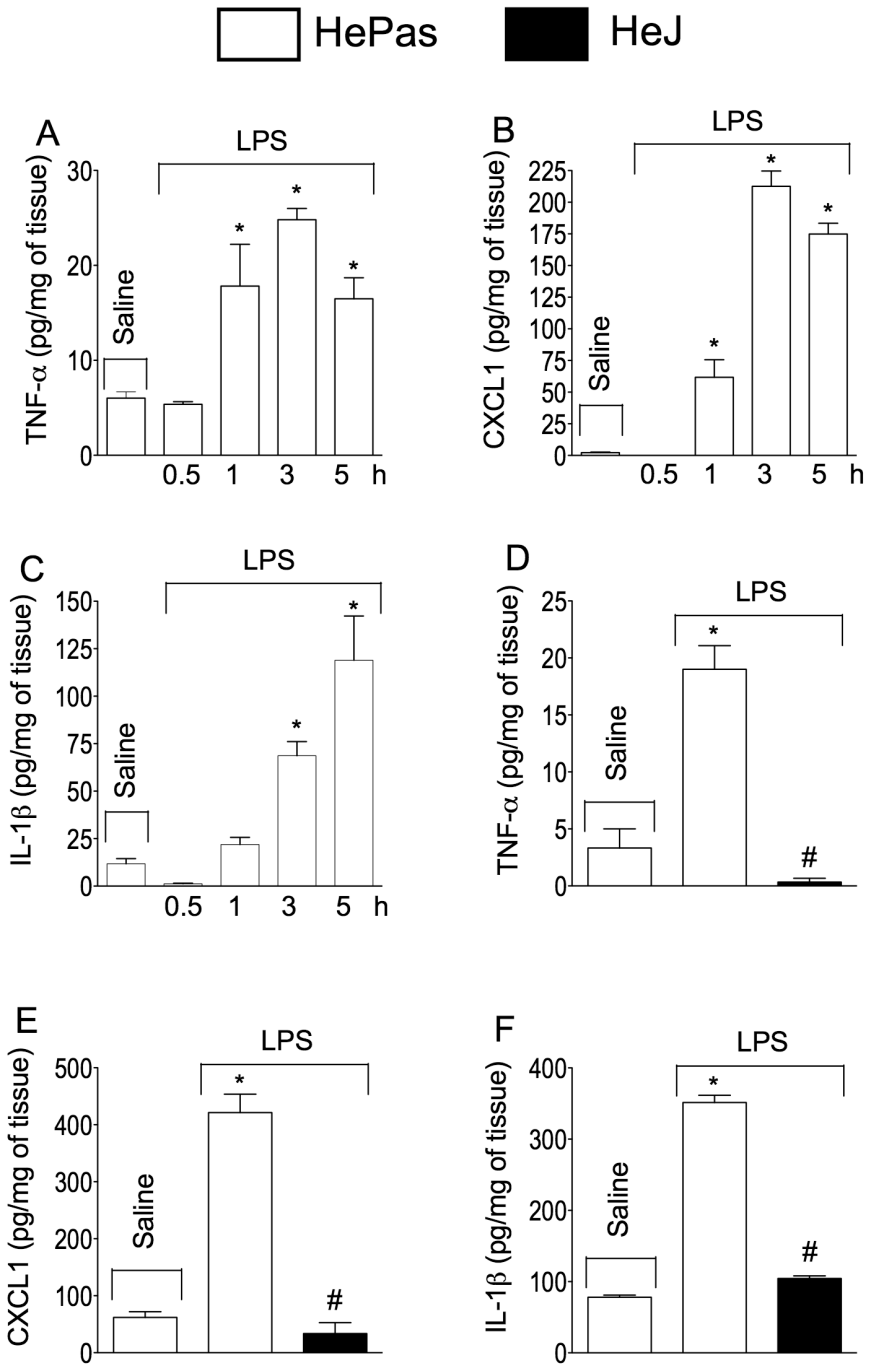
Cytokines production profile induced by LPS in the mice paw: role of TLR4. LPS (100 ng/paw) or saline was injected into the HePas mice hindpaw and subcutaneous tissue samples were collected at indicated time points for (**A**) TNF-α, (**B**) CXCL1/KC or (**C**) IL-1β determination by ELISA. LPS (100 ng/paw) or saline was injected into the HePas or Hej mice hindpaw. At 3 h after LPS injection, subcutaneous tissue samples were collected for (**D**) TNF-α, (**E**) CXCL1 or (**F**) IL-1β determination by ELISA. *P<0.05 compared with saline control; #*P*<0.05 compared with HePas injected with LPS. One-way ANOVA followed by Tukey's t test.

**Figure 6:**
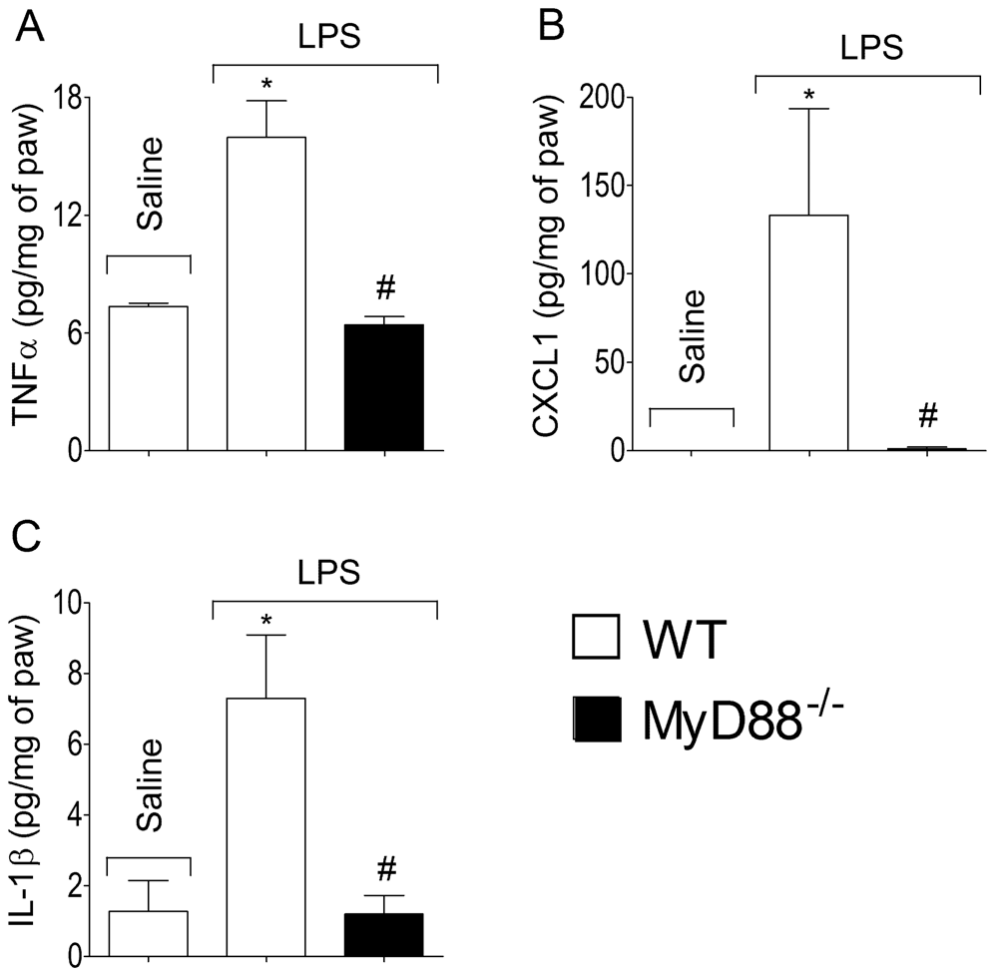
Pronociceptive cytokines production induced by LPS depends on MyD88 signalling. (**A**) MyD88^+/+^ (C57BL/6 background) and MyD88^-/-^ (C57BL/6 background) mice received i.pl. injection of LPS (100 ng/paw) or saline. At 3 h after LPS injection, the subcutaneous tissue samples were collected for (**A**) TNF-α, (**B**) CXCL1 or (**C**) IL-1β determination by ELISA. *P<0.05 compared with saline control, #*P*<0.05 compared with MyD88^+/+^ mice injected with LPS. One-way ANOVA followed by Tukey's t test.

To validate that they mediated hyperalgesia, their expression profiles were determined after mice paw LPS injection. In the plantar tissue, LPS induced a time-dependent increase from 1–5 h in TNF-α, IL-1β and KC/CXCL1 release (Figure 5A, B and C). Interestingly, TNF-α production was maximal between 1 and 3 h whereas KC/CXCL1 and IL-1β increases were delayed and peaked instead between 3 and 5 h (Figure 5A, B and C). On the other hand, in the TLR4 signalling mutant mice, at 3 h LPS injection failed to induce any increases in their release (Figure 5D, F and G). Moreover, in MyD88 null mice the same negative response occurred (Figure 6A, B and C).

### LPS induced a TNF-α-dependent production of CXCL1 and IL-1β

**Figure 7:**
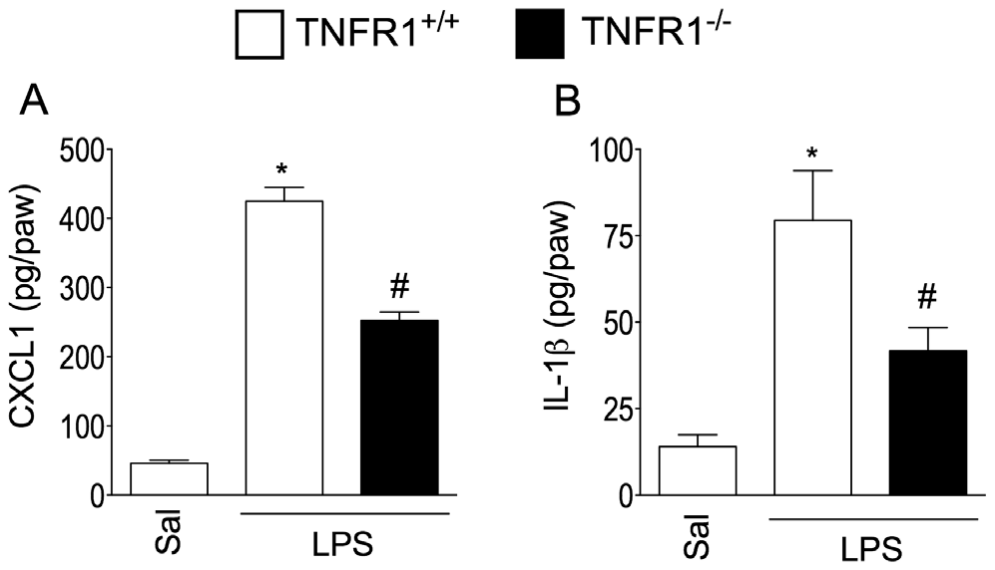
LPS induces a TNFR1-dependent production of CXCL1/KC and IL-1β. Saline (20 µl/paw) or LPS (100 ng/paw) was injected into the TNFR1^+/+^ (C57BL/6 background) or TNFR1^-/-^ (C57BL/6 background) mice hindpaw. At 3 h after LPS injection, the subcutaneous tissue samples were collected for (**A**) CXCL1/KC, (**B**) or (**C**) IL-1β determination by ELISA. *P<0.05 compared with saline control, #*P*<0.05 compared with TNFR1^+/+^ mice injected with LPS. One-way ANOVA followed by Tukey's t test.

Since increases in TNF-α release preceded rises KC/CXCL1 and IL-1β expression, we evaluated whether or not TNF-α/TNFR1 signalling activation leads to KC/CXCL1 and IL-1β release. In TNFR1 null mice, LPS-induced production of both cytokines in the paw tissue was reduced compared with levels identified in WT mice (Figure 7A,B).

### LPS induces myeloperoxidase activity increase through TLR4/MyD88 signalling pathway activation

**Figure 8:**
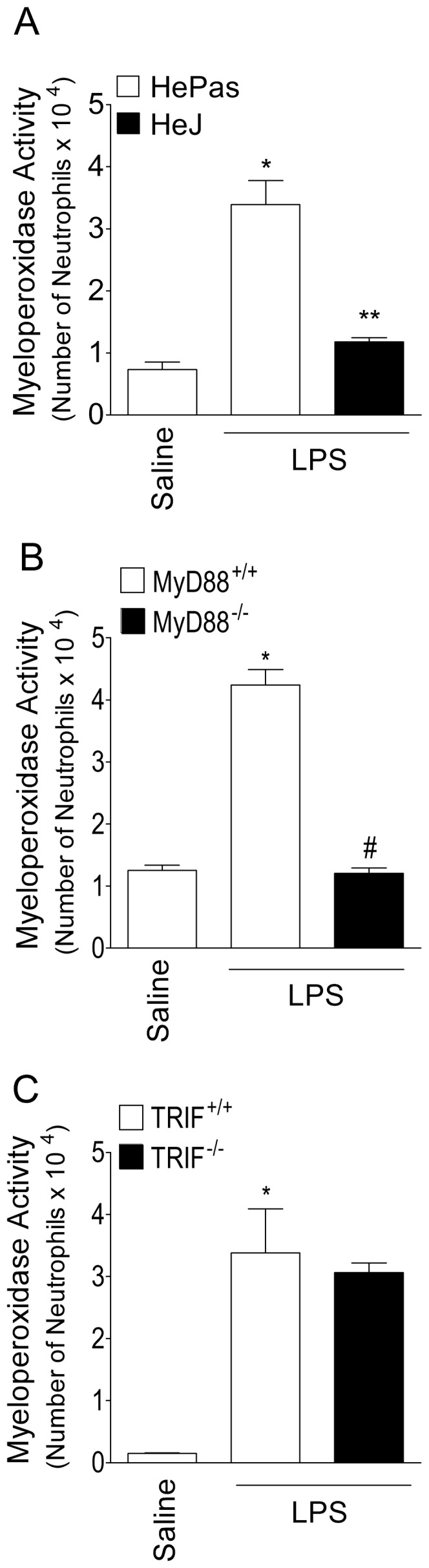
Role of TLR4, MyD88 and TRIF signalling pathways in LPS-induced increase of myeloperoxidase activity in the mice paw skin. **(A)** HePas and Hej mice received i.pl. injection of LPS (100 ng/paw) or saline (20 µl/paw). **(B)** MyD88^+/+^ (C57BL/6 background) and MyD88^-/-^ (C57BL/6 background) mice received i.pl. injection of LPS or saline. **(C)** TRIF^+/+^ (129Sv background) and TRIF^-/-^ (129Sv background) mice received i.pl. injection of LPS or saline. Myeloperoxidase activity was evaluated 5 h after LPS injection. *P<0.05 compared with saline control, #*P*<0.05 compared with HePas, MyD88^+/+^ or TRIF^+/+^ mice injected with LPS. One-way ANOVA followed by Tukey's t test.

In order to make a parallel between mechanical hyperalgesia and inflammation, the myeloperoxidase activity was evaluated. Paw edema was not used as inflammatory parameter because there is no measurable edema at the dose of LPS used in the present study (data not show). The increase of myeloperoxidase activity occurring in WT (HePas, C57BL/6 and SV129) mice was inhibited in HeJ (Figure 8A) and MyD88^-/-^ mice (Figure 8B) while it was unaltered in TRIF^-/-^ (Figure 8C) mice, respectively. These results suggest that TLR4/MyD88 rather than TLR4/TRIF-dependent signaling is solely responsible for eliciting LPS-induced increase of myeloperoxidase activity.

## Discussion and Conclusions

LPS-induced inflammation has been used as a model representing gram-negative bacteria-induced inflammation [6], [7], [8], [9], [10], [16], [17]. It may also represent the effect of endogenous molecules such as fibronection, hsp60, S100A8, S100A9 since they bind and signal through TLR4 [18], [19]. LPS-induced inflammation model presents advantages when microbicidal protective responses are not the aim of study as compared to live bacteria models in which controlling infection is an essential feature. On the other hand, live bacteria present additional hyperalgesic molecules besides LPS such as N-formylated peptides and αhaemolysin [20]. Therefore, every experimental condition has its contribution and accomplishes specific questions. In the present study, it was found that mouse plantar activation of TLR4 triggered by LPS induces mechanical inflammatory hyperalgesia solely through MyD88 dependent signaling pathway. TLR4 induced increases in TNF-α, KC/CXCL1, IL-1β, prostanoid and sympathetic amine release all contributed to eliciting this response.

TLR4 activation elicits responses in different tissues through MyD88-dependent MyD88-independent TRIF adaptor protein signaling pathways. For instance, both adaptor molecules are necessary for dendritic cell maturation [21]. On the other hand, in some other cell types activation of either one of these pathways solely accounts for TLR4 response control [14], [22]. MyD88 signaling activation by TLR4 involves recruitment and binding to this adaptor molecule. Their interaction leads to NF-κB activation and ultimately increases in cytokine release and enzyme expression, such as cyclooxygenase-2, that are involved in eliciting inflammation and pain [5], [23], [24]. TRIF-dependent signaling also activates NF-κB-dependent transcription although not as potently as that elicited through MyD88 dependent signaling [22]. TRIF-dependent signaling activates the transcription factor IRF (interferon regulatory factor)-3, which is responsible for anti-viral responses including increases in type I IFN release [14]. To effectively manage a clinical sign (such as pain) or disease, it is important to determine which of these pathways undergoes activation by TLR4 interaction with LPS. We found that LPS-induced mouse paw mechanical hyperalgesia depends solely on TLR4/MyD88 signaling since this response was inhibited in TLR4 mutant mice and MyD88^-/-^ mice, but not in TRIF^-/-^ mice. Thus, LPS-induced mechanical hyperalgesia, does not depend on TRIF adaptor molecule recruitment and activation by TLR4 indicating that it is unlikely that IRF-3-related genes are primarily involved in this hyperalgesic response. The resident paw skin cells involved in LPS-induced hyperalgesia might include macrophages, dendritic cells and mast cells in which there is MyD88-dependent and TRIF-independent LPS signaling [16], [25].

Although the lack of participation of TRIF in TLR4 signaling has been demonstrated in some other tissue types, there are no reports regarding its involvement in pain processing. Our rationale for assessing its involvement in mouse paw mechanical hyperalgesia is that in dendritic cells and bone marrow-derived macrophages, LPS induces TNF-α production through the TRIF rather than the MyD88 dependent signaling pathway [16]. TRIF-induces increases in TNF-α biosynthesis by promoting mRNA translation. This effect is dependent on MAPK-activated protein kinase 2 (MK2) stimulation by p38 [16]. As the TNF-α response to TLR4 activation is TRIF dependent in a mouse paw resident cell, the dependence of TLR4 induced mechanical hyperalgesia in the mouse paw on MyD88 activation was unexpected [3].

LPS induces NF-κB-dependent production of cytokines including TNF-α, KC/CXCL1 and IL-1β in vitro [26]. These cytokines are involved in carrageenin-induced mechanical hyperalgesia in mice [5]. Carrageenin induces the production of TNF-α and KC/CXCL1, which in turn induce the production of IL-1β, responsible for triggering prostanoid production. KC/CXCL1 also induces sympathetic amine-dependent hyperalgesia. Furthermore, it was observed that TNFR1 deficiency did not change the production of KC/CXCL1, suggesting that in carrageenin-induced inflammatory pain the production of KC/CXCL1 is not dependent on increases in TNF-α release [5]. This finding is consistent with the finding that suppressing KC/CXCL1 release did not inhibit TNF-α-induced hyperalgesia in mice [5]. However, in rats, inhibiting CINC-1/CXCL1 release instead inhibits TNF-α-induced hyperalgesia [27]. In the present study, LPS-induced activation of TLR4/MyD88 resulted in increases in TNF-α expression. Increases in KC/CXCL1 and IL-1β release are involved in LPS-induced mechanical hyperalgesia since suppressing activity of these cytokines inhibited this response. Furthermore, in TNFR1^-/-^ mice LPS-induced KC/CXCL1 and IL-1β release was reduced, which is consistent with the release/production profile of these cytokine after LPS challenge. In fact, LPS-induced paw TNF-α production that was initially detectable already at 1 h, but then peaked at 3 h and decreased thereafter. On the other hand, KC/CXCL1 and IL-1β production was delayed compared with TNF-α, suggesting that increases in TNF-α might promote KC/CXCL1 and IL-1β production.

The acute hyperalgesic effect of cytokines has been attributed to the production of prostanoids and sympathetic amines since cyclooxygenase inhibitors (e.g. indomethacin) and sympathomimetic blockers (e.g. guanethidine) inhibit the mechanical hyperalgesia induced by cytokines [28], [29], [30]. Herein, LPS-induced mechanical hyperalgesia was also inhibited by treatment with indomethacin and guanethidine suggesting the participation of prostanoids and sympathetic amines.

The dose of LPS was selected in a dose-response curve to evaluate mechanical hyperalgesia. At this dose, LPS does not induce measurable paw edema preventing the evaluation of an additional inflammatory sign together with hyperalgesia. Nevertheless, LPS induced an increase of myeloperoxidase activity in the paw skin. The myeloperoxidase activity is used as an indirect marker of neutrophil and macrophage counts in tissues [13], [31], [32], [33]. The LPS-induced increase of myeloperoxidase activity lined up well with the results on mechanical hyperalgesia since was also dependent on TLR4/MyD88 and independent on TLR4/TRIF signaling. The recruited neutrophils and macrophages contribute to inflammatory hyperalgesia by producing, for instance, PGE_2_ [32], [33].

This study raises the possibility that targeting MyD88 signaling without affecting TRIF signaling reduces LPS-induced hyperalgesia, but would at least not eliminate responses mediated by TRIF signaling activation. Therefore, developing different drugs that selectively target either LPS-induced TLR4 receptor activation and linked may improve management of pain and inflammation. For instance, quercetin is a flavonoid with an analgesic effect in inflammation models [34]. This drug inhibits LPS-induced production of inflammatory cytokines, MAP kinases and NF-κB activation by a mechanism related to inhibition of Src- and Syk-mediated PI3K- (p85) tyrosine phosphorylation and subsequent TLR4/MyD88/PI_3_K complex formation, which limits the activation of downstream signaling pathways [17]. Cell-permeable peptides and peptidomimetic agents are being developed that selectively inhibit MyD88 homodimerization and function [35]. Regarding TLR4/TRIF signaling, LPS treatment reduces H5N1 influenza virus replication and lethality in a TRIF-dependent manner [36]. Hence, a mixture of monophosphorylated lipid A species called MPL was recently approved by Food and Drug Administration as an adjuvant [37]. MPL preferentially induces TLR4/TRIF-dependent signaling with reduced inflammatory cytokine production compared to activation of TLR4/MyD88 signaling and at the same time it is useful as an adjuvant in vaccination [37]. TLR4/TRIF signaling is also important for clonal expansion in vaccination [38]. Therefore, selectively targeting or activating MyD88 or TRIF may provide a therapeutic option in, biological systems for to selectively reduce pain and inflammation without compromising TLR4-induced responses that are life sustaining. Furthermore, the present data suggest that LPS-induced mechanical hyperalgesia could be used to determine the activity of therapies targeting MyD88. Moreover, these results could also lead to a better understanding on how to develop a new vaccine formulation that causes less pain by targeting TLR4/MyD88 signaling.

In conclusion, the present results indicate that LPS-induced mechanical hyperalgesia does not depend on TLR4/TRIF signaling, but rather depends on LPS-induced activation of TLR4 receptor and recruitment of the adaptor molecule, MyD88, leading to increases in TNF-α, KC/CXCL1 and IL-1β release, which are responsible for inducing the production of prostanoids and sympathetic amines that sensitize the nociceptor resulting in the observed mechanical hyperalgesia. Moreover, in a different vein we have previously demonstrated in a carrageenin-induced inflammatory pain model in mice that LPS-induced hyperalgesia is dependent on TNF-α acting on TNFR1 to lead to increases in KC/CXCL1 and IL-1β release.
